# Effects of Nanotexture on Electrical Profiling of Single Tumor Cell and Detection of Cancer from Blood in Microfluidic Channels

**DOI:** 10.1038/srep13031

**Published:** 2015-09-16

**Authors:** Muhymin Islam, Mohammad Motasim Bellah, Adeel Sajid, Mohammad Raziul Hasan, Young-tae Kim, Samir M. Iqbal

**Affiliations:** 1 Nano-Bio Lab, University of Texas at Arlington, Arlington, TX 76019, USA; 2 Department of Electrical Engineering, University of Texas at Arlington, Arlington, TX 76011, USA; 3 Nanotechnology Research Center, University of Texas at Arlington, Arlington, TX 76019, USA; 4 Department of Interdisciplinary Studies and Department of Biology, University of Texas at Arlington, Arlington, TX 76011, USA; 5 Department of Bioengineering, University of Texas at Arlington, Arlington, TX 76010, USA; 6 Department of Urology, University of Texas Southwestern Medical Center at Dallas, Dallas, Texas, 75390, USA

## Abstract

Microfluidic channels have been implemented to detect cancer cells from blood using electrical measurement of each single cell from the sample. Every cell provided characteristic current profile based on its mechano-physical properties. Cancer cells not only showed higher translocation time and peak amplitude compared to blood cells, their pulse shape was also distinctively different. Prevalent microfluidic channels are plain but we created nanotexture on the channel walls using micro reactive ion etching (micro-RIE). The translocation behaviors of the metastatic renal cancer cells through plain and nanotextured PDMS microchannels showed clear differences. Nanotexture enhanced the cell-surface interactions and more than 50% tumor cells exhibited slower translocation through nanotextured channels compared to plain devices. On the other hand, most of the blood cells had very similar characteristics in both channels. Only 7.63% blood cells had slower translocation in nanotextured microchannels. The tumor cell detection efficiency from whole blood increased by 14% in nanotextured microchannels compared to plain channels. This interesting effect of nanotexture on translocation behavior of tumor cells is important for the early detection of cancer.

Circulating tumor cells (CTCs) are found in peripheral blood of many cancer patients^1^,^2^,^3^. Definite enumeration of CTCs can serve as an indicator of the effectiveness of therapeutic interventions and to estimate disease prognosis^4^,^5^,^6^. Exceedingly low numbers of CTCs, ranging 1-200 per milliliter (ml) of blood, makes it extremely challenging to detect them^7^,^8^,^9^,^10^,^11^. A number of cancer cell sorting techniques like centrifugation, chromatography, and fluorescence/magnetic-activated cell sorting have been employed, however, these are limited in yield, and purity^12^, and some of these require expensive optical equipment^5^,^13^,^14^,^15^. Microfluidic systems have emerged as fascinating platforms to detect cancer cells. Due to high specificity and selectivity, aptamers have been incorporated in several microfluidic setups to detect and enrich tumor cells^13^,^15^,^16^,^17^. However, aptamers are not available for all types of cancers. Their reproduction and faithful attachment to device surfaces is also composed of long and tedious processes. Therefore, label-free microfluidic isolation that does not require multiple additional “tags” or “labels” of rare cells is preferable^18^. The opportunity lies in using distinctive physical properties of CTCs such as density, adhesion, size, and deformability for label-free separation.

Several studies have demonstrated that cell capture, cell growth, adhesion and orientation are influenced by nanoscale topography of the surfaces^19^,^20^,^21^,^22^. In tissue engineering, studies have shown that nanostructured scaffolds can significantly increase densities of certain cells^23^,^24^. Some applications of textured surfaces are also found in biosensors, proteomics, and light emitting diodes^25^,^26^,^27^,^28^. Nanotextured surfaces can be prepared using processes like micro-contact printing, stencil assisted patterning, long polymer chemical etching etc. which are all time-consuming or cost-prohibitive^19^,^22^,^29^. Several studies have also reported plasma etching to prepare nanotextured surfaces^30^,^31^,^32^.

Recently, we reported single micropore device to detect tumor cells from whole blood at an efficiency of 70%^1^. The tumor cells exhibited characteristic electrical signals which distinguished the malignant cells from the other blood-based cells. Here, a microfluidic channel device with nanotextured walls is shown to detect metastatic renal cancer cells from mixture of whole blood based on their translocation behavior at 86% efficiency. The nanotexture resulted in added feature to slow down the translocation of more than 50% of tumor cells. Consequently, the added tumor cell detection efficiency came from fundamental cell-surface interactions which mimic cell-basement membrane interactions that occur during intravasation and extravasation. It is known that metastatic tumor cells depict large elastic deformations to pass through endothelial cell layers and basement membrane during these steps^33^.

The translocation mechanism of cells depends on applied fluid pressure, cell size, orientation of the cells, cellular and nuclear mechanical properties and interactions of cells with the surface of the device^5^,^14^,^34^. The mechanical rigidity of a cell is defined by its cytoskeleton components, which in turn is a function of cell health^35^,^36^. When a normal cell mutates to a malignant one, it undergoes reorganization of the cytoskeleton which leads to changes in mechanical properties of that cell. It has been reported already that many types of cancer cells not only have larger sizes than red and white blood cells (RBCs and WBCs), but also have different elasticity than healthy cells^5^,^14^,^34^.

The reported microdevice is simple, reusable and efficient. This scheme does not require fluorescent tags, surface functionalization or pre-processing of the blood except dilution for the detection of tumor cells. As cells pass through the microchannel, the physical blockage of the channel results into distinctive current pulses for different cells. Pulse magnitude depends on the cell size and pulse duration corresponds to the time required for the cell to pass through the microchannel. The translocation time depends on cell elasticity and cell-surface interactions in the channel. Pulse shape corresponds to the physical and mechanical properties of the cells. The tumor cells show distinctive translocation behavior because of their size, mechano-physical properties, and possibly due to their increased interactions with the nanotextured surfaces.

## Materials and Methods

All chemicals were obtained from Sigma-Aldrich (St. Louis, MO) unless noted otherwise. All the methods were carried out in *accordance* with the approved guidelines.

### Device Fabrication

**Figure 1:**
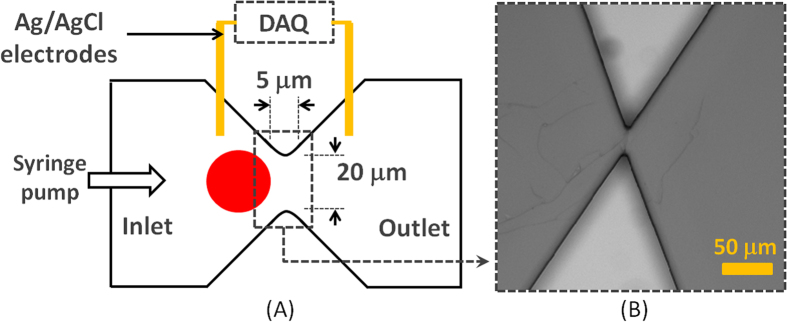
(**A**) Schematic diagram of the measurement setup. The syringe pump is connected to the inlet of microfluidic device. The Ag/AgCl electrode pair is connected to data acquisition system (DAQ) to record the data. (**B**) Optical micrograph of the microchannel.

Soft lithography was done to fabricate the PDMS device and micro-RIE was used to create nanotexture. The microfluidic device contained one inlet and one outlet connected via single microchannel. The dimension of the channel was 20 μm × 20 μm × 5 μm (width × height × length) (Fig. 1(A)). The layout of the device was first designed in *AutoCAD* and transferred to a glass photomask. The device was fabricated on a silicon wafer by spin coating SU-8 2010 (1000 rpm, 35 seconds) followed by photolithography. Next, the pattern was translated to PDMS which was mixed (10:1, wt/wt) with Sylgard 184 silicone elastomer curing agent (Dow Corning). This mixture was degassed in a desiccator for 1 h to get rid of the bubbles. Then PDMS was poured on the master and heated to 75 °C for 5 min and then 150 °C for 10 min. The PDMS mold was peeled off the master and fluidic ports were punched in PDMS mold. The mold was cleaned in isopropyl alcohol (IPA), rinsed in de-ionized water (DI water) and dried in nitrogen flow.

To create nanotexture, the channel surface was etched in reactive ion etch series 800 plasma system. The etching was performed using oxygen (O_2_) and carbon tetra fluoride (CF_4_) for 20 min. After etch, device was washed in sonicated IPA followed by dip in piranha solution (H_2_O_2_:H_2_SO_4_ in a 1:3 ratio). Glass slides, cleaned in piranha solution, were used to cover both nanotextured and plain PDMS devices. The PDMS devices with glass slides were treated with UV-Ozone plasma for 15 min and hermetically bonded together. The fabricated devices were filled with 1X PBS that contained 5 mM magnesium chloride (pH 7.5).

### Device Characterization

A confocal microscope was used to image the fabricated silicon master mold (Fig. 1(B)) and KLA-Tencor Alpha-Step IQ profilometer was used to measure heights of the features. Surface topography of PDMS and nanotextured PDMS surfaces were evaluated quantitatively using a Dimension 5000 atomic force microscope (AFM). The root mean square surface roughness was measured over scan area of 10 μm × 10 μm. Micrographs of PDMS samples were captured in the ambient air with 15% to 20% humidity at a tapping frequency of ~300 kHz. The analyzed field was measured at a scan rate of 1 Hz with 256 scanning lines.

### Measurement Setup and Optimization of the System

The measurement setup was very similar as it has been reported before^1^. Ag/AgCl electrodes were biased through a data acquisition card (National Instruments) to measure ionic current. The inlet of the microchannel was connected to a syringe pump (Harvard Apparatus) through a tubing adapter. The cells were suspended in phosphate buffered saline (PBS) and controlled fluid pressure was applied using the syringe pump to push cells through microchannel. Schematic diagram of the entire measurement setup is shown in Fig. 1(A). The ionic conductivity of the microchannel reduced due to physical blockage of the channel when cells passed. The microfluidic device consisted of single channel and the dimensions of the channel did not allow more than one tumor cell to travel through. It has been reported before that at a flow rate above 20 μl/min, the measurement system would miss some fast cell translocation events^1^,^37^. Additionally, to enhance the cell surface interactions lower flow rate is preferable. But lower flow rates, for example 5 μl/min or less, result into very low throughput. Considering all these constrains, a flow rate of 16.67 μl/min was used (1 ml/hour). A similar optimization was done for the sampling frequency of the electrical measurements^1^,^37^. As a first principle, at 16.67 μl/min, cells take 15–350 μs to pass through. These numbers translate to sampling frequency range of 0.002–0.04 MHz if we were to measure only one data point for each translocation event. The 0.2 MHz sampling frequency measured a data point after every 5 μs thus capturing 3 data points for the fastest cell (15 μs) event and 70 data points for a slowest cell (350 μs). Hence the current sampling interval was set at 5 μs (0.20 MHz) and the applied bias voltage was 5 volts.

### Human Derived Primary Renal Cancer Cell Culture and Collection of Rat Blood

Metastatic renal tumor cells were isolated from the brain tissue of consenting patient at the University of Texas Southwestern Medical Center at Dallas, Texas, USA as per the approved Institutional Review Board protocol^38^. The cell dissociation and collection has been reported before^39^. These cells were known metastatic renal cancer cells that had metastasized to the brain of the patient.

The blood samples were collected from tail of a rat by restraining the animal. The blood was collected in tubes with K2-EDTA as anticoagulant.

## Results and Discussion

### Surface Topography of Nanotextured Substrates

**Figure 2:**
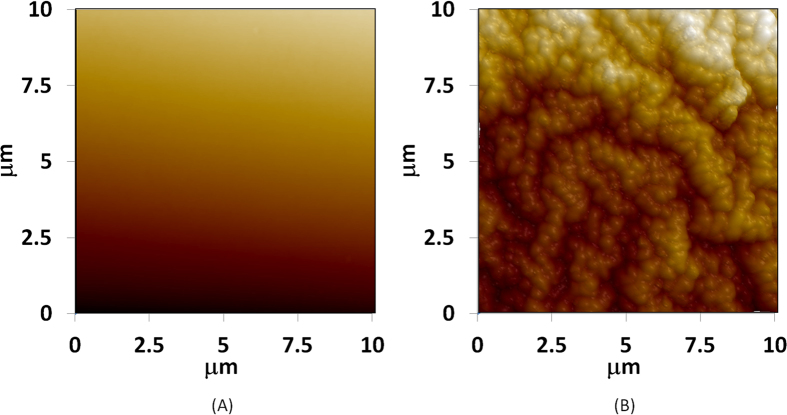
AFM micrographs of (**A**) plain and (**B**) nanotextured PDMS surfaces. Nanotexture was achieved with O_2_: CF_4_ plasma etch.

The measured average roughness of plain and nanotextured PDMS surfaces were19.95 ± 9.17 nm and 519.17 ± 103.71 nm, respectively. The AFM micrographs of plain and nanotextured surfaces are shown in Fig. 2.

### Translocation Behavior of Tumor Cells through Plain and Nanotextured Microchannels

**Figure 3:**
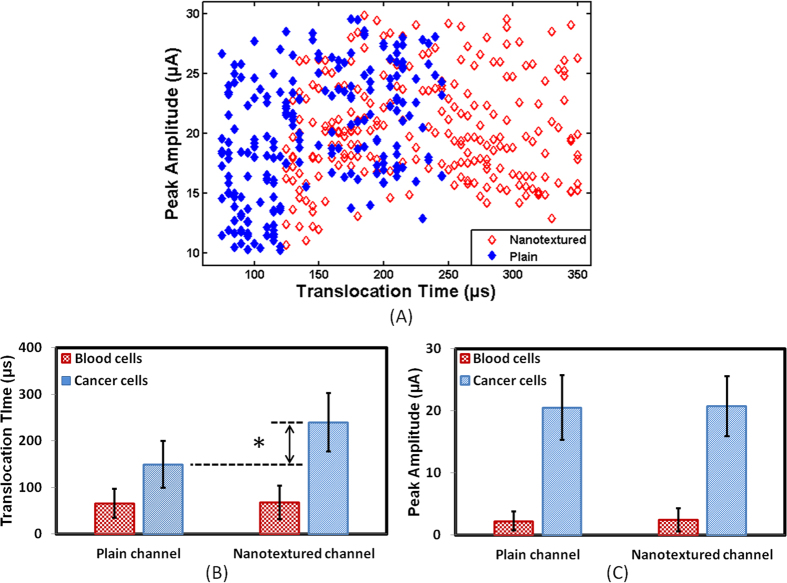
Translocation behavior of renal cancer cells through plain and nanotextured microchannels (n ≈ 200). (**A**) Scatter plot shows data for tumor cells through plain channel (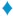) and nanotextured channel (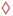). (**B**,**C**) show the averages of pulse peak amplitudes and pulse widths (translocation time) through the two types of channels (* p-value < 0.01).

The tumor cells were suspended in PBS at a concentration of 1000 cells/ml. These cells were passed through plain and nanotextured microchannels for 10 min. The experiments were repeated at least 5 times. All the cancer cells gave distinct pulses. The values of average current peaks for metastatic tumor cells were not statistically different between two types of devices. For plain and nanotextured microchannels, average current peaks were 20.53 ± 5.21 μA and 20.76 ± 4.81 μA, respectively. The average translocation time was higher in nanotextured microchannels compared to plain microchannels. For plain and nanotextured microchannels, average translocation times were 149.23 ± 49.99 μs and 239.85 ± 62.30 μs, respectively. More than 50% tumor cells took longer to pass through the nanotextured microchannels compared to plain microchannels (Fig. 3(A)) and the average translocation time increased by ~60% (Fig. 3(B)). Current peak depended on the physical blockage of the channel and amount of blockage depended on cell size and orientation. As the cell sizes were same for both devices the current peak was same (Fig. 3(C)). On the other hand, the translocation time showed difference which stemmed from the cells’ interactions with the nanotextured channel surfaces.

Nanotopographic surfaces have various impacts on cell functions^40^,^41^,^42^,^43^,^44^,^45^,^46^,^47^. Nanotextured topography offers biomimetic cell-stimulating cues very similar to nanotextured interfaces *in vivo*. Basement membranes of most tissues are composed of complex mixtures of nanoscale structures^40^. In our experiments, cells sensed nanotopography and reacted by bridging or conforming in a selective manner. The nanoscale patterning is known to significantly impact the organization and type of focal adhesions either by disrupting their formation or by inducing specific integrin recruitment^41^. As integrins are directly linked to the nucleus, the response of cells to the nanoscale topography may have also resulted in altered gene expression. Upon adhesion to a substrate, the cells are known to probe their environment and move using nanoscale processes like filopodia and lamellipodia. Filopodia probe the environment and their ends serve as anchor points for cell movement^41^. Cells also interact with the topography by deforming membrane and by modifying the functioning of cell surface. These factors may all be inducing significant impacts on mechanical properties of cells such as stretching, spreading, elasticity etc^40^,^41^,^42^,^43^,^44^. Nanotextured surfaces also influence the volume and shape of the nucleus and increase the elastic modulus of the cells^42^,^43^,^44^. The translocation time of a cell fundamentally depends on the cell elasticity and cell-surface interactions^37^, but the cumulative effects of aforementioned facts might have all contributed to the increased translocation time of tumor cells in nanotextured microchannels. Statistical analysis of translocation time and peak amplitude for tumor cells in plain and nanotextured microchannels were performed using one-way analysis of variance (ANOVA). The peak amplitude did not show appreciable statistical difference (*p*-value > 0.7) but translocation time was significantly different between two types of microchannels (*p*-value < 0.01).

### Translocation Behavior of Blood Cells in Plain and Nanotextured Microchannels

Trans-location behavior of blood cells was also characterized through plain and nanotextured microchannels. Blood cells were diluted 100 times in 1X PBS and processed through the microchannels for 5 minutes. The experiments were repeated at least three times and the results through the two types of channels were similar (Fig. 3(B)). The RBCs are smaller compared to WBCs, thus a single RBC would be very small to create significant detectable pulse. The microchannels would miss many of the RBCs. The average peak amplitudes for blood cells in plain and nanotextured microchannels were 2.23 ± 1.53 μA and 2.43 ± 1.85 μA, respectively and average translocation times were 66 ± 30.65 μs and 67.74 ± 36.07 μs, respectively. Thus, for blood cells, the average translocation time and average peak amplitude were not significantly different between plain and nanotextured microchannels. Only 7.63% blood cells had slightly higher translocation time in nanotextured microchannel compared to plain channel and the average translocation time increased by 2.63%. The nanotexture thus did not show increased affinity towards blood cells. As a result, translocation behavior remained very similar in both microchannels for majority of the blood cells. Statistical analysis of translocation time and peak amplitude in plain and nanotextured microchannels was performed using ANOVA. Statistical difference of translocation time and peak amplitude in two devices was not significant for blood cells (*p*-values > 0.05).

### Pulse Shapes of Tumor and Blood Cells

**Figure 4:**
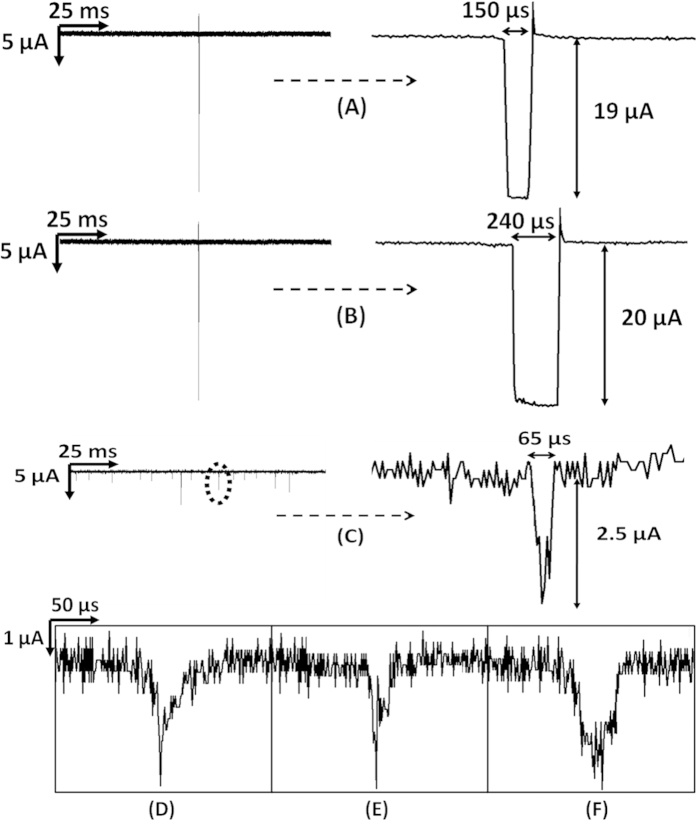
Representative electrical profile of the translocated cells through the microchannel, (**A**) cancer cell through plain microchannel, (**B**) cancer cell through nanotextured microchannel, (**C**) blood through nanotextured PDMS microchannel, close-up of electrical pulse profiles of blood cells through (**D**,**E**) plain and (**F**) nanotextured PDMS microchannels.

Pulse shapes of cancer cells were very uniform (Fig. 4(A)). Due to deformable nature, pseudopod formation, and interactions with the surface, tumor cells had higher translocation time and ripples of very small magnitude were observed at the bottoms of inverted bell shaped pulses. This is again related to the ionic current stability during the translocation of tumor cells. The pulse bottoms did not show fluctuations like those seen in blood cell pulses. The uniform pulse shape confirmed that only one cancer cell passed through the microchannel at a time and the ionic current was uniformly blocked. The sampling interval of the data acquisition system was 5 μs. The average translocation times for tumor cells in plain and nanotextured microchannels were 149.23 μs and 239.85 μs, respectively. Hence, in plain and nanotextured microchannels a pulse for one tumor cell contained 30 and 48 data points, respectively. Each current value thus corresponded to a cross-sectional contour depicting the physical dimension of the whole cell^1^. The translocation data gave enough information to precisely track the three dimensional electrical profile of the tumor cells. From Fig. 4(A) it can be seen that for cancer cells, the pulse width is more in nanotextured microchannel and also higher number of ripples can be seen at the bottom of the pulse. The plausible explanation can be the formation of pseudopods in the nanotextured channel. The interactions of tumor cells were higher with nanotextured surfaces and cells created higher number of pseudopods in nanotextured microchannel^19^. The formation of pseudopod resulted change in shape of tumor cells and affected their translocation profile. It was also observed that the current rose slightly at the end of every pulse of tumor cells in both types of channels. It was transient and current stabilized in very short period of time (20 μs). This transient might have been due to sudden rush of ions under the applied bias to equilibrate the potential across the channel. For blood cells, some pulses had several spikes which indicated that more than one cells, most probably RBCs, passed together. The diameters of WBCs vary within a range of 5-16 μm. The larger WBCs showed deeper pulses but the bottom of the pulses had sharper angles (Fig. 4(C)) as blood cells were stiffer and did not create any pseudopod. Figure 4(D–F) show close-up electrical profile of blood cells. These pulses had multiple spikes or depicted irregular shapes, in contrast to the pulses of cancer cells. At the sampling rate of 5 μs, in both channels on an average, a pulse contained approximately 13 data points of current for a blood cell. Thus, the pulse shapes can depict the physical dimension of blood cells as well. Statistical analysis of translocation time and peak amplitude for blood and tumor cells showed significant differences between both microchannels (*p*-value < 0.01).

### Discrimination of Tumor and Blood Cells

**Figure 5:**
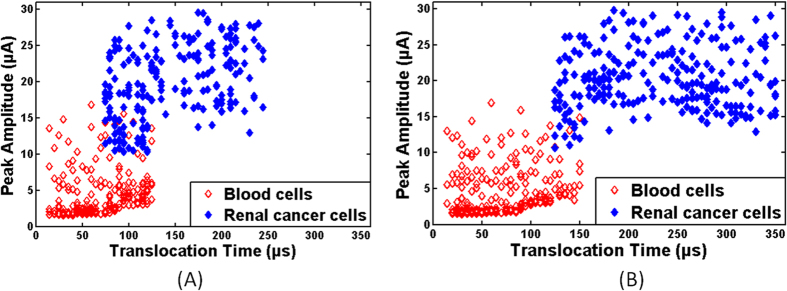
Representative translocation data of blood and renal cancer cells through (**A**) plain and (**B**) nanotextured microchannels (n ≈ 200).

The pulse data for blood and renal cancer cells through plain and nanotextured microchannels is shown in Fig. 5. In plain microchannels, the peak amplitude and translocation time for blood cells were 16.81 μA and 125 μs, respectively. On the other hand, lowest peak amplitude and translocation time for renal cancer cells in the plain microchannels were 10.29 μA and 75 μs, respectively. Thus, there was an overlapping region in translocation behavior for both types of cells in plain microchannels. The calculations showed that 82% tumor cells exhibited distinctive behavior in plain microchannels and remaining 18% of tumor cells were enveloped in the translocation region of blood cells. Similarly, in nanotextured microchannels, the peak amplitude and translocation time for blood cells were 16.95 μA and 150 μs, respectively. On the other hand, lowest peak amplitude and translocation time for renal cancer cells in nanotextured microchannels were 10.73 μA and 125 μs, respectively. In nanotextured microchannels, 93% of tumor cells were discriminated from blood cells based on their translocation time and peak amplitude. Translocation time of tumor cells was higher in nanotextured microchannels as discussed above and consequently the discrimination efficiency was improved.

### Detection of Tumor Cells from Blood

**Figure 6:**
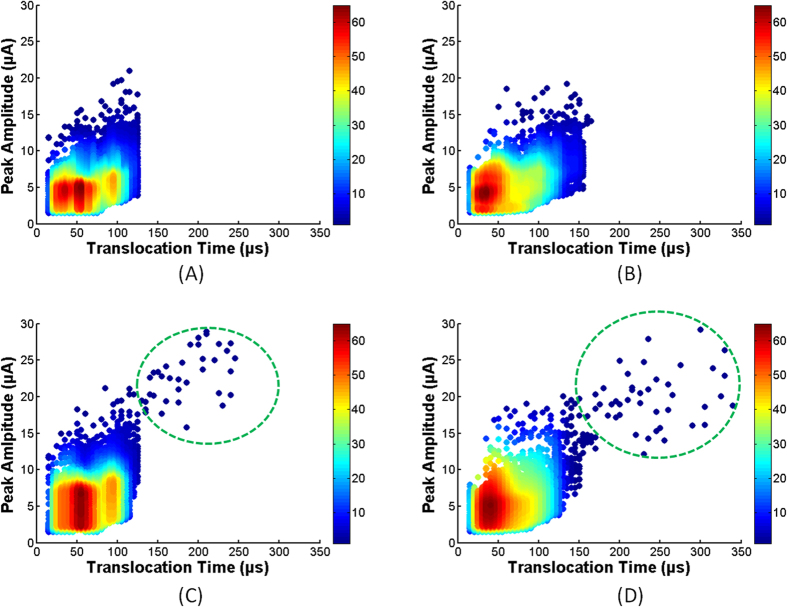
Data plots for blood cells through (**A**) plain and (**B**) nanotextured microchannels; data plots for cancer cells spiked in blood through (**C**) plain and (**D**) nanotextured microchannels. The typical regions of detected cancer cells are enclosed in dotted green circle.

The aim of the microfluidic device was to detect cancer cells from blood and other bodily fluids. To achieve that target the blood was diluted ten times with 1X PBS. The dilution of blood made the process easier by reducing the density and viscosity of the blood^1^. This diluted blood solution was passed through the microchannels at a flow rate of 16.67 μl/min. The experiments were done with each type of device for 10 min and repeated twice. The recorded translocation data of blood cells from both plain and nanotextured devices were used for density plot as shown in Fig. 6(A), respectively. The color variation represents the densities of cells. For plain and nanotextured channels, the peak amplitudes were 19.2 μA and 21.04 μA, respectively and the maximum translocation times were 125 μs and 165 μs, respectively. It should be noted that the blood cells shown in Fig. 3(B) were diluted 100 times. Here these were diluted 10 times. As the dilution factor decreased from 100 to 10, the upper threshold for blood cells increased slightly due to possible clumping at high concentration.

Next, the renal cancer cells were spiked in diluted blood (dilution factor was 10) at a concentration of 100 cells/ml. This suspension was run through microchannels for 15 min. The experiment was repeated twice. In both runs, the flow rate was kept same as before. Thus the total volume of processed sample was 0.5 ml and the total number of cells to be passed through the microchannel was 50. The density plots of the pulse data from tumor cells in blood are shown in Fig. 6(C) for plain and nanotextured microchannels, respectively. Tumor cells can be discriminated in plain microchannels by comparing Fig. 6(A). The data comparison between Fig. 6(B) for nanotextured microchannels also shows clear distinction of cancer cells. Typical translocation regions of renal cancer cells, obtained from Fig. 5, are shown by green dotted circle in Fig. 6(C). Most of the cells showed familiar peaks in both channels. The majority of the cancer cells exhibited higher translocation time and peak amplitude to distinguish them from blood cells. But for both devices several cells fell in a region which was very close to blood cells. These small percentages (5.56% in plain microchannels and 4.65% in nanotextured microchannels) of cells were discriminated by observing pulse shape very closely. In plain microchannel, cancer cells were detected with an efficiency of 72% while in nanotextured microchannel 86% efficiency was achieved. Enhanced cell-surface interactions in nanotextured channel thus appreciably increased the detection efficiency of tumor cells.

## Conclusions

Microfluidic device was fabricated to detect cancer cells from whole blood based on their distinctive translocation behavior. The surface nanotexture of the microfluidic channel retarded the metastatic renal cancer cells’ translocation by 50%. This interesting phenomenon increased tumor cell detection efficiency by 14% in nanotextured microchannel compared to plain channel. There may be possibility of false positives or false negatives, especially if there are larger-than-average blood cells or smaller-than-average tumor cells. This limitation can be overcome by observing the pulse shape closely. But more rigorous approach can be obtained to distinguish tumor cells by considering their biochemical properties. To improve the efficiency and sensitivity ligands such as antibodies or aptamers can be incorporated in the device. Using multiple devices in parallel can increase the throughput. Nanotextured microchannel devices can also be implemented to distinguish various types of cells identify cancer cells at different stages such as metastatic and non-metastatic tumor cells.

## Additional Information

**How to cite this article**: Islam, M. *et al.* Effects of Nanotexture on Electrical Profiling of Single Tumor Cell and Detection of Cancer from Blood in Microfluidic Channels. *Sci. Rep.* **5**, 13031; doi: 10.1038/srep13031 (2015).
